# A matched pair cluster randomized implementation trail to measure the effectiveness of an intervention package aiming to decrease perinatal mortality and increase institution-based obstetric care among indigenous women in Guatemala: study protocol

**DOI:** 10.1186/1471-2393-13-73

**Published:** 2013-03-21

**Authors:** Edgar Kestler, Dilys Walker, Anabelle Bonvecchio, Sandra Sáenz de Tejada, Allan Donner

**Affiliations:** 1 Epidemiological Research Center in Sexual and Reproductive Health (CIESAR), Guatemala City, Guatemala; 2 Department of Global Health and Obstetrics and Gynecology, University of Washington, Seattle, USA; 3 National Institute of Public Health (INSP), Cuernavaca, Mexico; 4 Department of Epidemiology and Biostatistics and Robarts Clinical Trail, Robarts Research Institute, Western University, London, Canada

**Keywords:** Maternal morbidity, Perinatal mortality, Intervention package, Matched cluster trial, Indigenous

## Abstract

**Background:**

Maternal and perinatal mortality continue to be a high priority problem on the health agendas of less developed countries. Despite the progress made in the last decade to quantify the magnitude of maternal mortality, few interventions have been implemented with the intent to measure impact directly on maternal or perinatal deaths. The success of interventions implemented in less developed countries to reduce mortality has been questioned, in terms of the tendency to maintain a clinical perspective with a focus on purely medical care separate from community-based approaches that take cultural and social aspects of maternal and perinatal deaths into account. Our innovative approach utilizes both the clinical and community perspectives; moreover, our study will report the weight that each of these components may have had on reducing perinatal mortality and increasing institution-based deliveries.

**Methods/Design:**

A matched pair cluster-randomized trial will be conducted in clinics in four rural indigenous districts with the highest maternal mortality ratios in Guatemala. The individual clinic will serve as the unit of randomization, with 15 matched pairs of control and intervention clinics composing the final sample. Three interventions will be implemented in indigenous, rural and poor populations: a simulation training program for emergency obstetric and perinatal care, increased participation of the professional midwife in strengthening the link between traditional birth attendants (TBA) and the formal health care system, and a social marketing campaign to promote institution-based deliveries. No external intervention is planned for control clinics, although enhanced monitoring, surveillance and data collection will occur throughout the study in all clinics throughout the four districts. All obstetric events occurring in any of the participating health facilities and districts during the 18 months implementation period will be included in the analysis, controlling for the cluster design. Our main outcome measures will be the change in perinatal mortality and in the proportion of institution-based deliveries.

**Discussion:**

A unique feature of this protocol is that we are not proposing an individual intervention, but rather a package of interventions, which is designed to address the complexities and realities of maternal and perinatal mortality in developing countries. To date, many other countries, has focused its efforts to decrease maternal mortality indirectly by improving infrastructure and data collection systems rather than on implementing specific interventions to directly improve outcomes.

**Trial registration:**

ClinicalTrial.gov,http://NCT01653626.

## Background

Efforts to decrease maternal mortality in developing countries worldwide are well documented and mortality ratios have decreased with national and international efforts, but unacceptably high ratios persist [1,2]. Maternal and perinatal morbidity and mortality are major problems for which progress has been inadequate in Guatemala [1,3]. The Millennium Development Goals (MDGs) aim to reduce child and maternal mortality throughout the world [4] and compel governments to increase their involvement solving this problem. If progress continues at the present pace, MDG achievement in the Central American region is unlikely for decades. Analyses of key strategies to decrease maternal and perinatal mortality clearly point to the presence of a skilled attendant at birth and access to emergency obstetric care as crucial evidence-based interventions [5,6]. Successful programs acknowledge that these two interventions are not mutually inclusive.

Over the past years, the Guatemalan government completed a project funded by the International Bank for Reconstruction and Development (IBRD) of the World Bank, focused on the construction of clinics with 24-hour functional labor and delivery units for uncomplicated vaginal deliveries without need for an operating room or blood supply (*Centro de Atención Permanente*, or CAP) [7]. In the districts under study the number of CAPs increased from 11 to 68 between 2006 and 2010, providing sorely needed infrastructure changes. However, building the population’s confidence in clinic-based care, improving the quality of care, and expanding the types of services provided, were not addressed by the IBRD project. Our study seeks to build on the IBRD’s successful infrastructure improvements and address the urgent need for both increasing the proportion of births in medical facilities and improving emergency obstetric care in those clinics.

### Study rationale

In countries like Guatemala, the majority of births in rural indigenous areas are at home and attended by a non-professional. Women with complicated labor often face four major delays: recognizing complications; deciding to seek care; reaching a health facility due to lack of transportation or resources; and lastly, receiving appropriate care at the facility [8]. Successful and effective interventions used in other countries to reduce the maternal mortality ratio (MMR) have focused on the development and strengthening of health institutions with trained personnel and improving access to timely and suitable emergency obstetric care [9]. Our trial will implement a package of strategies aiming to address known weaknesses: inadequate provision of emergency obstetric care, low utilization of clinic-based services, and poor links between TBAs and weak linkages between TBAs and the formal health care system.

#### Inadequate provision of emergency obstetric care

Our study will be conducted in districts having the highest MMRs in Guatemala: 260 deaths per 100,000 women in Alta Verapaz, 240 in Huehuetenango, 164 in Quiche and 106 in San Marcos [10]. Although maternal mortality results from a complex interplay of clinical, cultural, socioeconomic and political issues, the majority of direct obstetric deaths are preventable with timely and effective EmOC [11,12]. For example, opportune management of pre-eclampsia/eclampsia is believed to be sufficient to reduce maternal mortality rates to 50 per 100,000 women without the need for advanced technology and life support mechanisms [13]. Improved EmOC is essential for reducing maternal and perinatal mortality in our intervention districts, where it is currently poor, or simply unavailable.

#### Low utilization of clinic-based obstetric care

Perinatal and maternal outcomes are both highly dependent on having skilled care available when needed. If emergency care is only available at clinics, births occurring in health facilities are an important indicator for improved access to essential care [14]. Despite the recent improvements in infrastructure, the majority of women in our study districts continue to prefer childbirth with TBAs or traditional midwives: in 2009 only 6% of pregnant women in Huehuetenango, 26% in Quiche, 34% in Alta Verapaz and less than 5% in San Marcos delivered in health facilities [15]. Internationally, there are numerous explanations for women’s low or untimely utilization of professional medical care, especially in poor rural communities. Important factors include the lack of information regarding the availability of health services/providers, lack of control of household resources and decision making authority, inadequate or absence of transportation, and absence of or poor linkages between health centers and communities [16]. Fear of medical treatments, condescending attitudes and poor treatment by providers, refusal of spouses to permit care, disrespect for modesty, perceptions of poor quality of care, limited hours of service and language constraints are other reasons documented in the literature in Guatemala [17]. Strategies that aim to address these barriers are, and have been, introduced however in general little rigor is used in measuring the effectiveness of such strategies.

#### Weak linkages between traditional birth attendants (TBAs) and the formal health care system

The relationship of Guatemalan TBAs with the health care system has evolved over the years. TBA-related interventions in Guatemala have historically focused on one of three areas: training of TBAs, integrating TBAs into public hospitals and most recently, the provision of culturally-appropriate services by including TBAs as health providers in clinics. Myriad institutions both local and foreign have provided training and support to TBAs; however, the results of these efforts have been mixed [18-20]. Furthermore, there are many gaps in the literature as far as how TBAs have been trained, methods used and actual program results. A formalized and effective referral system between TBAs in the community and institution-based care in clinics is a key element particularly to deliver timely emergency obstetric care [8].

A general limitation of the literature is that many interventions include some form of TBA-clinic linkage; as well as other components such as TBA training, financial assistance or transportation support, making it difficult to tease out the impact of TBA linkage alone [21]. However, given the comprehensive approach of our intervention package, the results of other experiences from similar settings are still highly applicable; for example, TBA linkage interventions targeting specific geographic areas with high MMRs with special attention paid to remote rural areas [22]. Additionally, most studies focus on mortality outcomes rather than proportion of institution-based births, our main outcome of interest and an index indicator of decrease community maternal morbidity and mortality. Increased institutional birth will presumably result in less complicated home deliveries, and greater access to adequate emergency care. Since the ultimate goal of reducing perinatal mortality is achievable through skilled attendance at birth by an appropriately trained and supported health worker with access to basic and comprehensive EmOC [23], increasing the proportion of institution-based births is a valid indicator of progress toward mortality reduction.

There is convincing evidence that specific interventions are effective in controlled settings, but more rigorous implementation trials and cost-effectiveness studies are needed in settings such as rural Guatemala [24]. Similarly, increasing community demand for obstetric care through community mobilization activities is strongly recommended, particularly with increasing levels of community participation and ownership; however further evaluation is needed on the cost-effectiveness, sustainability, and scalability of these approaches. One example, the Behavior Change Interventions for Safe Motherhood program aimed to reduce mortality by increasing the timely use of key maternal and neonatal health interventions: improving and ensuring high-quality clinical services and skills, creating a locally appropriate mass media component to help define safe motherhood as a broad social issue and establishing community mobilization systems to effect change where pregnant women and their families live [25]. The Safe Motherhood message aimed to make maternal health both a community effort and an explicitly shared responsibility. At program completion, 55% of the exposed women gave birth at a medical facility — an increase from 31% at baseline — compared to only 31% of unexposed women.

Our study is also important because it will be carried out where few other interventions have been implemented. Exceptions include the Casa Materna project in Huehuetenango [26] and the Mother Care and Calidad en Salud initiatives in San Marcos [27,28]. Of the very few impact evaluations of interventions implemented in Guatemala [29] none have used a randomized clinical trial design, the gold standard for measuring impact. Even among the descriptive papers of implemented projects there is little follow-up reporting, making it difficult to know if any of the introduced changes had sustained impacts on either process our outcome indicators.

A number of recent reviews find that a comprehensive package of interventions spanning the continuum of care from the home to the hospital, with a robust community mobilization component, has the potential to significantly improve perinatal outcomes including the proportion of births occurring in health facilities [23,29,30]. Nevertheless, to our knowledge, no randomized control trial has been carried out to evaluate the effectiveness of a package of interventions specifically directed at improving maternal and perinatal health. The premise of the study is that this package of three interventions — each directed at one of the three problem areas outlined above — will increase institution-based delivery and decrease perinatal mortality in the four districts with the highest MMRs in Guatemala. The study is also expected to improve the measurement of our outcomes of interest among both intervention and control facilities in all four districts.

## Methods/Design

This matched pair cluster randomized trial will assess the effectiveness of a package of three interventions that together aim to decrease perinatal mortality by increasing the proportion of institution-based deliveries: 1) implementation of PRONTO, a simulation-based program for team training and effective management of obstetric and perinatal emergencies; 2) application of a social marketing strategy to increase utilization of public sector health facilities for childbirth; and 3) strengthening the link between TBAs and public sector health services through the participation of professional midwives. The unit of randomization and the unit of analysis will be at the CAP level.

### Sampling methods

The intervention package’s target population is primarily indigenous pregnant Guatemalan women and newborns that live in the catchment areas of the intervention CAPs, as well as clinicians and nurses working in those CAPs and receiving obstetric and neonatal emergency training. In order to assess and control for clinic variability, an inventory and infrastructure survey was first conducted in each of the existing 68 clinics in the study districts. This survey served to assess which clinics met inclusion criteria and provided the data for matching.

#### Inclusion criteria

All 68 CAPs in the four districts were eligible to participate. All communities have TBAs. Each CAP is open 24 hours per day, has personnel (physicians, professional nurses, nurses assistants, educators) and the basic equipment necessary for performing normal vaginal and emergency identification and stabilization. To be included, on average, seven births per month had to have occurred (during the 6 months prior to sample selection) at CAPs and they must be located less than 6 hours by vehicle from the district’s commercial center. Clinics without 24 hours services and those with operating rooms were excluded from the trial. 38 clinics were excluded by these criteria leaving 30 participating clinics.

#### Sample size

The perinatal mortality rate (PMR) was the primary outcome of interest informing our sample size calculation. Due to the wide variability in PMRs and the expected poor quality of existing data with likely underreporting across the four study districts, we decided to use baseline data from Alta Verapaz, the district with the largest number of participating CAPs, to calculate the standard deviation in PMRs. This choice was also justified by our interest to use districts as the primary matching factors; the district selected with the largest number of clinics, was expected to have the largest intra-district variation. Therefore, for our final sample size calculation we used the mean PMRs for all four districts (estimated to be 52 deaths per 1,000 live births according to available baseline data) and the standard deviation across clinics for Alta Verapaz. With a two-sided significance level of 0.05 and a desired power of 80%, a sample size of 15 matched pairs is sufficient to detect an expected 25% reduction in mean PMRs in the intervention group. This sample size is conservative since statistical power will be increased through the use of mean change scores from baseline as the actual outcome measure.

### Randomization and matching procedure

During the pretrial inventory and infrastructure survey, baseline data including volume of deliveries, PMR, and proportion of clinic-based delivery versus home delivery was collected and used for matching.

**Figure 1:**
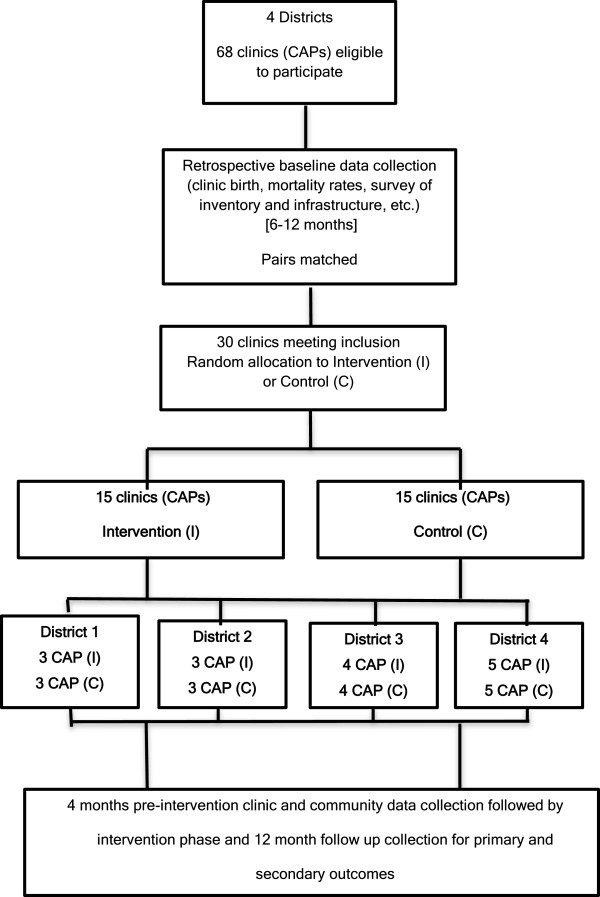
Flow diagram of perinatal intervention package trial phases.

We matched the 30 CAPs in pairs using the following criteria in the order in which they appear: by district, by low or high PMR within a given district and by low or high volume of live births. For the last two criteria we defined low as below the median and high as equal or higher than the median. For random assignment to each of the experimental units within pairs, we randomly selected one “district” within each pair and then allocated it to the intervention or control group. As shown in Figure 1, this process resulted in 15 intervention clinics and 15 control clinics.

Given our inability to strictly control patterns of movement of women and infants between clinics, the study design includes another important step: as part of the matching process we selected CAPs that are geographically separated in such a way that it would be highly unlikely that a woman would travel to an intervention site rather than a closer facility. However such travel will be thoroughly monitored to every possible extent so as to be able to measure the resulting degree of possible contamination, if any, and take this into account in the data analysis and interpretation of results. The monitoring procedure will note the communities of residence of women delivering in the clinics. Furthermore, the study’s conservative sample size requirement will allow detection of an intervention effect even if it is mildly diluted by such experimental contamination.

Clinic supervisors and staff will not be blinded to their allocation group as they will see evidence of the social marketing campaign and be trained in the intervention group. They will remain blinded to the results until the end of the study to alleviate potential tensions that could rise in case they wish to introduce aspects of the intervention to control group CAPs. We will also emphasize to all supervisors and staff that no conclusions can be drawn regarding the possible benefit of the intervention until the study is completed.

### Description of interventions

Three interventions will be jointly implemented in the randomly selected study CAPs. The first component seeks to directly impact perinatal mortality by improving EmOC, while the second and third components are designed to reduce PMRs by increasing the proportion of institution-based births (Table 1).

1) Emergency obstetric and perinatal training of clinicians with PRONTO^a^

The first component of the package to be implemented is the PRONTO training program, a high-fidelity low-tech in-situ simulation-based obstetric and perinatal emergency and team-training program developed In Mexico in 2008–2009, and subsequently piloted in four hospitals [31]. Focusing on inadequate EmOC as a key obstacle to women’s survival, the PRONTO program is a novel approach to training in the diagnosis and treatment of the most deadly and common obstetric emergencies and basic neonatal resuscitation, within the limits of available resources. The curriculum was designed to be multi-disciplinary, based on the latest evidence-based thinking regarding critical emergency care and is grounded in team training and communication theories. The program uses a low-tech, low-cost simulator (PartoPants TM) and all materials included in the simulations are based on the actual materials (medications, instruments, equipment) available at the particular site.

Results from the pilot study in Mexico were positive [31], with hospital-based goals achieved, evidence-based practices (i.e., active management of the third stage of labor and magnesium sulfate for preeclampsia/eclampsia) implemented and teamwork and self-efficacy measures improved. PRONTO training also aims to identify and overcome latent errors in care — errors related to system problems, such as facilities, purchasing of supplies or equipment and staffing.

Specific training activities are carried out during two modules, a total of 14 hours of training. Module I imparts team-training, and simulation in obstetric hemorrhage and neonatal resuscitation. Module II, 8 hours, 2–3 months later, reviews the previous module training and introduces simulation in preeclampsia/eclampsia and shoulder dystocia.

We adapted the PRONTO curriculum for use specifically in the Guatemalan primary care clinic setting. In addition to adding content on retained placenta and chorioamnionitis, PRONTO in Guatemala will include activities designed to help health care providers reflect on identity and culture, and use those reflections to make concrete improvements to their care of indigenous women during labor and delivery.

2) Social marketing campaign to promote childbirth in clinics

Across countries, “the most obvious competition to a normal, healthy pregnancy and birth remain cultural, ritual and social values” [32]. Taking into consideration the complex and varied factors that prevent women from using the health system for obstetrical care, our trial will promote the advantages of institution-based delivery and the quality of care provided there, for rural Guatemalan women. Social marketing is the systematic application of marketing concepts and techniques to achieve specific behavioral goals for a social good; [33] it can be applied to promote merit goods or to make a society avoid demerit goods and thus to promote society’s well being as a whole [34]. Although social marketing has been used in some health related areas such as smoking prevention, condom promotion and road traffic safety, evidence is scarce regarding its application to institution-based obstetric care. Nevertheless, social marketing strategies apply because they seek to influence people’s behaviors by focusing on consumers’ preferences. In this context we aim to enhance the interface between home- and clinic-based care and through this campaign encourage women to deliver in clinics.

Programs that succeed in changing behavior are often based on scientific theories of behavior change and the results of formative research conducted prior to implementation, as strategies rooted in this kind of research tend to make them more culturally acceptable and effective. Formative research, in the form of a systematic review, will identify the main determinants of factors that could potentially enhance the care-seeking behavior of pregnant women and the information gap that needs be collected during this phase. The formative research will be conducted to understand preliminary target audience attitudes, knowledge and practices in relation to institution-based childbirth. Identifying the target audience, goals and objective will allow us to specify the marketing strategies to be applied and inform the subsequent qualitative and quantitative data collection phase. The qualitative component will consist of focus groups and individual interviews carried out with key informants in selected districts to assess attitudes, perceptions and barriers to institution-based childbirth.

Once we better understand the factors underlying the resistance to clinic-based childbirth, we can use different channels to promote its advantages.

With this information we will identify a list of possible audiences, learn about previous efforts to address low utilization of clinic-based care and identify salient issues. Thus, the formative research will help determine which type of communication channel (radio, television, community meeting, etc.) will be directed toward which audience (pregnant women, TBAs, partners of pregnant women, etc.) and which is likely to have the greatest acceptance and audience’s reach.

The design of the marketing strategy will be based on the main principles of social marketing strategy development: product (care-seeking behavior), promotion (channels, type of message), price (cost/barriers of institution-based deliveries) and place (home versus clinic) [35]. The strategy development phase aims to specify the problem, the final target audience, the scope of the campaign and behaviors and cultural factors to be addressed; only then will potentially effective marketing strategies emerge. For example, providing elements of traditional birthing environments in clinics seems promising, but what is the most effective strategy to let people know this is available; and more importantly, make them want to use the clinic instead of their own traditional birth environment at home.?

Prior to the launch of the social marketing campaign, baseline data will be collected to evaluate community knowledge regarding institutional based delivery. A sample of pregnant women living nearby, and at intermediate and far distances from clinics in the intervention communities will be interviewed at baseline and again ten months later after the campaign launch to assess exposure to communication channels and messages. These data will help assess the exposition to the audience to the campaign and the potential impact it might have in the number of deliveries occurring in district clinics.

3) Introduction of professional midwife liaison between TBAs and clinics

The third component of the trial will aim to strengthen TBA linkage with the formal health care system through the participation of professional midwives. It is widely recognized that in settings like rural Guatemala, TBAs are and will continue to be important providers of pregnancy and childbirth care [36,37]. Dozens of studies have examined TBA training, but important questions remain unanswered. For example, in terms of the effects of referral systems on the personal economy of TBAs; since they charge for their services only after delivery, why would they refer apparently normal, uncomplicated pregnant women to formal health care facilities?

Furthermore, training TBAs to improve their knowledge of obstetric emergencies is not sufficient to reduce mortality rates; establishing mechanisms to foster positive relationships with the formal health care system is essential. The traditional lack of coordination and interaction between TBAs and the health system can be addressed by defining and developing partnerships with TBAs. From a practical standpoint, there are multiple ways to link TBAs with formal health services. The WHO Department of Making Pregnancy Safer [37] suggests that the following roles for TBAs within the health system, when developed in conjunction with TBAs, are potentially effective:

•Advocate for maternal and newborn health needs.

•Encourage and even accompany women to essential antenatal and postnatal care visits and encourage them to have skilled care during labor and delivery.

•Support women and newborns in self-care and care compliance (nutrition, treatment, supplementation, immunization, scheduled appointments, birth and planning, family planning, infant feeding, etc.).

•Disseminate health information in the community and to families.

•Provide social support during and after birth, either as a birth companion or in the form of support to the household while the mother is away during childbirth.

•Serve as a link between women, families and communities and local authorities and formal health services.

**Table 1:** Mechanisms of intervention package in reducing perinatal mortality

**Problem**	**Contributing factors**	**Intervention**	**Objective**	**Result**	**Impact**
Poor perinatal outcomes including high mortality	Insufficient capacity to deliver effective emergency perinatal care in clinics	PRONTO training	Better prepare professional health care providers for obstetric and neonatal emergencies	Improve emergency obstetric care	Reduce perinatal mortality
	Lack of coordination and interaction between TBAs and health system	Professional midwife liaison	Strengthen the relationship between communities/TBAs and formal health services	Increase the proportion of institution-based births	
	Multidimensional factors preventing women from using the health system for obstetrical care	Social marketing campaign	Influence care-seeking behavior by promoting the advantages of institutional delivery		

Two professional midwives, with experience and good communication skills will be in charge of implementing this intervention and supporting these TBA activities. Beyond the roles suggested by WHO, the professional midwives will conduct a survey to assess the activities and practices of TBAs in the districts of study. Questions like the number of deliveries assisted per month, the number of pregnant women being followed, the number of emergency obstetric references made in the last year, the number of training session during the last year, etc. will help to determine ‘Active TBA’, defined as those who assisted at least one delivery per month in the last year. Once identified ‘Active TBA’ and the professional midwives will have a close and personnel relationship and meet to discuss cases of pregnancy and delivery complications. Training simulation-based in direct causes of maternal death will be developed. TBA with close relationships with CAPs will be invited to participate in PRONTO training. Moreover, after each training simulation-based session a simple assessment, hands up, will be obtained from the session. This simple method will be allowed to identify the knowledge, attitude and practice from TBA for direct maternal death causes. The professional midwives will also provide promotional material to TBA to encourage them to bring women to the CAP for vaginal delivery prior to complications.

We expect this strategy to result in a stronger, more positive relationships between TBAs, communities and formal health services, which we hope will impact the proportion of women giving birth in clinics, especially in emergency situations.

### Activities in control CAPs

No external intervention is planned for control clinics, although enhanced monitoring, surveillance and data collection will occur at all facilities during the trial. We will carefully monitor control facilities and their catchment areas to limit the risk of contamination. Contact with referral hospitals and outcomes of referred cases will also be carefully considered, as we anticipate that in both intervention and control facilities complicated cases will be transferred to the same referral hospitals. Additionally, we hope to provide all clinicians involved in labor and delivery with PRONTO training upon study completion and the messages and material developed in the social marketing component.

### Main outcomes and measures

This implementation project aims to directly impact perinatal health (Table 1), thus the primary outcome is the change in mean PMR (deaths occurring between 28 weeks gestation to 7 days, per 1,000 live births) in intervention versus control CAPs. Process outcomes expected to contribute to reduce PMRs (via PRONTO training) include increased knowledge and self-efficacy for diagnosis and initial management of common emergencies and self established clinic goal achievement for improving care. Intermediate outcomes expect to evaluate the exposure of the audience to the social marketing campaign will be change in attitudes, knowledge and practices in relation to institution-based childbirth among pregnant women and health personnel. The other main outcome measure (expected to be impacted primarily by the social marketing campaign and TBA linkage activities) is the change in proportion of institution-based deliveries in intervention versus controls CAPs. However, attributing impact to the various elements of the intervention will not be straightforward and will require careful analysis including additional qualitative work at the completion of the study.

### Data collection, management and storage

Data collection will occur in 3 phases: 1) pre-intervention (4 months), including qualitative work to drive the social marketing campaign, and quantitative work that will establish more reliable baseline data for morbidity and mortality indicators; 2) intervention, including collection of training and social marketing process indicators, and 3) post-intervention, including monthly follow up (12 months) in clinics and communities.

Data will be obtained from a variety of sources during the pre-intervention and implementation phases at three levels: 1) community level data to determine accurate denominators in terms of overall number of births and perinatal and maternal deaths, 2) clinic level data to be collected for all included CAPs, both intervention and control for morbidity and mortality indicators and 3) data to be collected only at intervention clinics, primarily related to participants in PRONTO training. On a monthly basis, trained personnel in each district will visit all offices responsible for registering certificates of live births, and stillbirths. Information from civil registry offices and health district offices will be compared to guarantee the quality of data collected and avoid the possible sub-registration typical of remote rural areas in developing countries. Community field workers will collect local home birth and death data from community liaisons and key informants. Finally, data on clinic deliveries in the study sites and their outcomes will be collected.

All field workers will be trained in data collection procedures including the ethical treatment of data. All data will be initially collected with paper forms from the aforementioned registries and offices and be assigned individual codes to maintain individual anonymity, confidentiality and privacy. Once the needed data is extracted from the file all individual identifiers will be removed, leaving only unique codes designating clinic and patient. These then will be entered into a computerized database; a separate log to match records will be kept in a secure locked file. Paper files will also be secured in a locked file accessible only to authorized personnel trained in ethical management of confidential data. Data quality and validity will be assured through bi-monthly data entry and queries to check outlying observations and key measures.

The instruments used to collect information from CAPs will be adapted from the near miss approach used by the WHO to extract appropriate data from patient records [38]. We will collect data for all normal and complicated deliveries including cases of maternal hemorrhage, preeclampsia, sepsis, referral and prolonged stay as well as neonatal complications including perinatal death, referrals and prolonged stay. Data related to individual events at the clinic level will be collected on an ongoing basis throughout the study at intervention and control sites. All information from individual charts for complicated cases will be extracted using an instrument that removes all individual identifiers and will be stored in a locked file cabinet.

### Data analysis plan

The effect of the intervention will be measured as the difference between matched clinics (intervention clinic minus control clinic) in PMR change from baseline to 12 months. Negative differences will indicate lower PMRs in the intervention group, adjusted for baseline rate. A one-sample two sided *t* test with corresponding confidence interval will be used to determine whether the mean rate difference as calculated over all matched pairs is statistically different from zero. A nonparametric permutation test will also be used with an exact p-value computed using the R statistical package. We will follow the same approach for secondary outcomes on which we have appropriate baseline data.

To adjust the mean rate difference in PMR between groups for variables that show some residual imbalance after pair-matching, a multiple linear regression model will be used, with the rate difference between matched clinics as the dependent variable and variables with baseline differences between matched clinics as the independent variables. Adjusted interferences for the effect of the intervention will be estimated by calculating the intercept of the resulting regression equation and comparing it to its estimated standard error [39].

### Ethical considerations

The trial design requires no individual-level outcome data given that the unit of analysis will be at clinic level. All needed data will be extracted from civil registries and institutional records without any patient identifiers. Since no information will be obtained directly from patients, no patient interviews are required. Given the aggregated nature of the study outcome measures, as well as the impracticality of seeking informed consent for patients whose clinical records will be reviewed, we sought and received waiver for consent from the Guatemalan Independent Latin Ethics Committee.

The project has already been reviewed and approved by the WHO Research and Ethics Review committee, the Independent Latin Ethics Committee, the Guatemalan National Committee for Ethics in Health, Ministry of Public Health, and the University of Washington IRB, Seattle. Overall, three main ethical issues have emerged: 1) the target communities are primarily indigenous populations, 2) pregnant women and adolescents will be exposed to elements of the intervention and 3) gender related issues may be at play in determining where pregnant women deliver. Firstly, since the intervention will be implemented in indigenous areas where the majority of women deliver outside a clinic setting, women who are influenced to deliver in facilities because of the intervention may be exposed to practices in the clinics that are both foreign and in contrast to the local traditional birthing practices. This important issue will be addressed during provider training. Secondly, pregnant women, and in particular pregnant adolescents will be exposed to the intervention and may or may not be empowered to make their own decisions about where to receive care. The intervention will not impact adolescent empowerment directly but the professional midwife liaison will be trained to be especially sensitive to the issues unique to pregnant adolescents and their families. Finally, potential gender related issues will be explored during the formative research phase in order to guide social marketing strategies and define the role of the professional midwife liaison.

## Discussion

A unique feature of this protocol is that we are not proposing an individual intervention, but rather a package of interventions which is designed to address the complexities and realities of maternal and perinatal health within the Guatemalan health system. The package addresses weaknesses in obstetric and perinatal care specific to the rural Guatemalan setting, and focuses on increasing skilled attendance at birth while simultaneously improving the quality of emergency obstetric and perinatal care in those clinics. Although interventions geared to increase skilled attendance at birth and improve EmOC quality have been implemented separately, we have found no reports of rigorously designed implementation projects aimed at jointly addressing these two critically interconnected issues. Our approach is further enhanced by a strong social marketing campaign, which is to our knowledge from published literature the first campaign of its type developed and implemented in countries with high maternal mortality ratios and also seek to improve the relationship of the TBA with the health system.

To date, Guatemala, like many other Central American countries, has focused its efforts to decrease maternal mortality indirectly by improving infrastructure and data collection systems rather than on implementing specific interventions to directly improve outcomes. This project aims to build on the strategies implemented by the IBRD and the government, moving beyond infrastructure and information systems to implement an intervention package to improve access and quality. This is an ambitious challenge, but has the potential to directly impact maternal and perinatal health, moving a step closer to achieving MDG4 and MDG5.

## Endnotes

^a^PRONTO course details are available for review at http://www.prontointernational.org

## Abbreviations

CAP: Centro de Atención Permanente (24-hour clinic); MDG: Millennium Development Goals; PMR: Perinatal Mortality Rate; MMR: Maternal Mortality Rate; TBA: Traditional Birth Attendant; IBRD: International Bank for Reconstruction and Development; WHO: World Health Organization

## Competing interests

The authors declare that they have no have competing interests.

## Authors’ contributions

EK initially conceived of the intervention package trial and drafted the original intention letter. Both EK and DW participated in all phases of protocol and manuscript production. AB researched and designed the social marketing component of the trial. SST conducted literature reviews and designed the data collection instruments. AD contributed to the design and supervised the sample size determination and data analysis plan and will carry out data analysis for the final evaluation. EK took the lead in drafting this manuscript, read and approved by all the other authors.

## Authors’ information

EK is the Director of the Epidemiological Research Center in Sexual and Reproductive Health (CIESAR) in Guatemala City. DW is a visiting professor at the National Institute of Public Health of Mexico (INSP) and an Associate Professor in Global Health and Obstetrics and Gynecology at the University of Washington, Seattle. AB is a researcher at the Department of Community Nutrition at the INSP. SST is a consultant for CIESAR. AD is a Professor in the Department of Epidemiology and Biostatistics of the University of Western Ontario and Director of its Biometrics/Robarts Clinical Trials division of the university’s Robarts Research Institute.

## Pre-publication history

The pre-publication history for this paper can be accessed here:

http://www.biomedcentral.com/1471-2393/13/73/prepub
